# Unmet need for family planning among married women in sub-Saharan Africa: a meta-analysis of DHS data (1995 – 2020)

**DOI:** 10.1186/s40834-022-00198-5

**Published:** 2023-01-11

**Authors:** Million Phiri, Clifford Odimegwu, Chester Kalinda

**Affiliations:** 1 Demography and Population Studies Programme, Schools of Public Health and Social Sciences, University of the Witwatersrand, Johannesburg, South Africa; 2 Department of Population Studies, School of Humanities and Social Sciences, University of Zambia, Lusaka, Zambia; 3 University of Global Health Equity, Bill and Joyce Cummings Institute of Global Health, KG 7 Ave., Kigali Heights, 5Th Floor, PO Box 6955, Kigali, Rwanda; 4 School of Nursing and Public Health, Department of Public Health, University of KwaZulu-Natal, Howard College Campus, George Campbell Building, Durban, 4001 South Africa

**Keywords:** Prevalence, Unmet need, Family planning, Meta-analysis, Sub-Saharan Africa

## Abstract

**Background:**

Closing the gap of unmet needs for family planning (FP) in sub-Saharan Africa remains critical in improving maternal and child health outcomes. Determining the prevalence of unmet needs for family planning among married women in the reproductive age is vital for designing effective sexual reproductive health interventions and programmes. Here, we use nationally representative data drawn from sub-Saharan countries to estimate and examine heterogeneity of unmet needs for family planning among currently married women of reproductive age.

**Methods:**

This study used secondary data from Demographic and Health Surveys (DHS) conducted between January 1, 1995 to December 31, 2020 from 37 countries in sub-Saharan African. An Inverse Heterogeneity model (IVhet) in MetaXL application was used to estimate country and sub-regional level pooled estimates and confidence intervals of unmet needs for FP in SSA.

**Results:**

The overall prevalence of unmet need for family planning among married women of reproductive age in the sub-region for the period under study was 22.9% (95% CI: 20.9–25.0). The prevalence varied across countries from 10% (95% CI: 10–11%) in Zimbabwe to 38% (95% CI: 35–40) and 38 (95% CI: 37–39) (I2 = 99.8% and *p*-value < 0.0001) in Sao Tome and Principe and Angola, respectively. Unmet needs due to limiting ranged from 6%; (95% CI: 3–9) in Central Africa to 9%; (95% CI: 8–11) in East Africa. On the other hand, the prevalence of unmet needs due to spacing was highest in Central Africa (Prev: 18; 95% CI: 16–21) and lowest in Southern Africa (Prev: 12%; 95% CI: 8–16). Our study indicates that there was no publication bias because the Luis Furuya-Kanamori index (0.79) was within the symmetry range of -1 and + 1.

**Conclusion:**

The prevalence of unmet need for FP remains high in sub-Saharan Africa suggesting the need for health policymakers to consider re-evaluating the current SRH policies and programmes with the view of redesigning the present successful strategies to address the problem.

**Supplementary Information:**

The online version contains supplementary material available at 10.1186/s40834-022-00198-5.

## Introduction

Attainment of universal access to sexual and reproductive health (SRH) services including family planning and achieving the Sustainable Development Goals (SDGs) SDG 3 and 5 requires deliberate and concerted policy efforts [1]. Thus, addressing factors and challenges such as sexual and reproductive health issues, unintended pregnancies, high fertility and unsafe abortions among women in the reproductive age should remain a priority [2, 3] Earlier studies by Ahmed et al. [4] and Ajayi et al. [5] have suggested that improved maternal health outcome is highly dependent on health care visits, rising demand, access, and uptake of contraceptives. Although several women in developing countries especially in sub-Saharan Africa (SSA) understand the importance of family planning (FP), many have unmet needs for FP [6, 7].

Family planning (FP) is pivotal in the improvement of maternal and child health [8] and return-on-investment strategies [9]. Following the 2012 London Summit on FP and the FP2020 initiative by Bill and Melinda Gates Foundation, the UK Department for International Development (DFID), the United States Agency for International Development (USAID), and the United Nations Population Fund (UNFPA) whose target is to reach 120 million new users of modern contraceptives in developing countries by the year 2020 [10, 11], there has been a revival in the needs for FP. However, the success of the FP2020 has not been attained partly due to the inability to track annual progress and delayed uptake of modern contraceptives [10] thus, increasing the gap in unmet needs for FP in developing countries [7, 12]. Calls to strengthen FP programmes in SSA to reduce unmet needs have been on the increase [13] as this influences regional and country-level decision making in attaining SDG 3.7.1. Therefore, estimating country level and regional prevalence of unmet needs for family planning would be key in evaluating country level FP programmes and interventions to guide policymakers on how to improve maternal and child health through improved resource allocation and redesigning of existing programmes.

Determining the prevalence of unmet need for family planning among women in the reproductive age is critical in measuring progress towards improving maternal and child health. There has been progress in understanding the factors that influence family planning [14–16], however unmet needs especially at the sub-regional level remains a challenge [7, 17]. Several earlier studies have determined unmet needs among young women [18], sex workers [19] and HIV positive women [20, 21]. Several studies on the unmet need for family planning have been published in other parts of the world. Many of the studies in SSA have focused mainly on examining country level prevalence and factors associated with unmet need for FP but neglected analyses of country and regional variations. At both country and sub-regional level, many factors such as socio-economic characteristics, religious beliefs, cultural beliefs, fertility norms, health behavior and prior SRH interventions can explain variations of the prevalence of family planning indicators [22]. However, a pooled analysis of the prevalence of unmet needs for FP using nationally representative data would be essential in providing a holistic regional picture as well as sub-regional differences. Data on regional variations is important to guide the identification and documentation of countries with best practices on SRH policy and programming. This data is useful to inform re-designing and implementation of maternal health programs in countries where the problem still exists. Here, we apply meta-analysis to determine the pooled prevalence and examine heterogeneity of unmet needs for family planning in SSA based on country level latest nationally-representative Demographic and Health Surveys (DHS) (collected between 1995 and 2020) from 37 countries. Furthermore, data from meta-analysis would be useful to support generation of evidence-based information to guide sharping SRH policy and programme design and implementation at country and sub-regional levels [23, 24].

## Methods

### Data sources

The data analysed in this study were extracted from the country latest Demographic and health survey (DHS) collected between 1995 and 2020 in 37 SSA countries. The datasets are publicly available from the DHS website https://dhsprogram.com/ [25]. The surveys are periodical nationally and population-based comprising large sample sizes which may vary between 5000 and 30,000. To enable comparisons among countries and sub-regions, DHS uses a standard sampling design using probability proportional to size when determining country level sample size. The DHS collate data using standardized questionnaires comprising: a household questionnaire, a women’s questionnaire, a men's questionnaire and the biomarker questionnaire. Furthermore, DHS surveys usually use two-stage stratified, (cluster), random sampling designs which involve the selection of enumeration areas in stage one and random selection of households in the second stage. In all selected households, women in the reproductive age 15–49 years are eligible to participate while men aged 15–59 years are also eligible to participate. Details on data collection and sampling methodology used by DHS are described elsewhere [25].

### Study selection and inclusion criteria

**Fig. 1:**
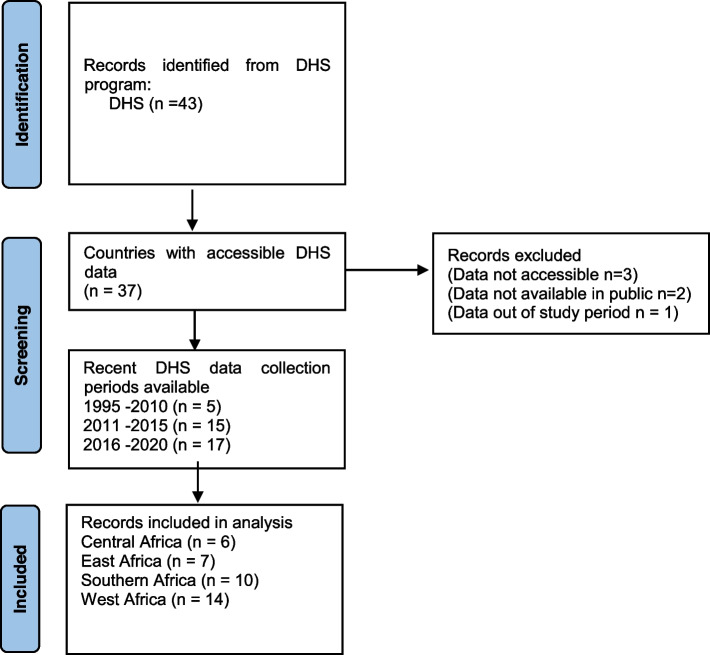
PRISMA diagram showing the selection and exclusion of DHS data

All sub-Saharan African countries with available DHS (1995–2020) and comprehensive data on the prevalence of unmet needs for family planning were included in this study. Countries with the latest DHS from 1994/5 to 2020 were eligible to capture the trend in unmet needs for family planning in SSA. Furthermore, including data in this period was used to give a holistic picture of unmet needs for family planning in SSA considering that some countries may be without the most recent DHS datasets. The selection criteria of countries to be included was summarized in the Preferred Reporting Items for Systematic Reviews and Meta-Analysis (PRISMA) diagram presented in Fig. 1. PRISMA is an evidence-based set of standard procedure to guide process of determining inclusion and exclusion criteria for reporting systematic and meta-analyses [26]. The sub-regional classification of countries in SSA is based on the United Nations (UN) geoscheme classification for SSA.

### Data extracted

Data sourced from the DHS program website included DHS datasets for 37 countries in sub-Saharan Africa conducted in the period 1995 -2020. The information extracted from the country-level datasets included the name of the country, year of DHS implementation, weighted country samples of married women 15–49, number of women who needed family planning, number of women with unmet need for FP, number of women with unmet need for spacing, number of women with unmet need for limiting and names of sub-regions where each country belong (Table 1). The outcome variable for the study, Unmet Need for family planning was defined as the number of women currently married or in union who are fecund and desire to either terminate or postpone childbearing, but are not currently using a contraceptive method [25].

**Table 1:** Distribution of women of reproductive age (15–49) with family need in sub-Saharan Africa 1995—2020

**Country**	**DHS Year**	**Weighted sample size**	**Number of women who need FP**	**Number of married women with unmet need for FP**	**Number of married women with unmet for Spacing**	**Number of married women with unmet for limiting**	**Data Source**	**Unit of analysis**	**Sub-Region**
Angola	2016	7,957	4,114	3,024	2,077	947	DHS	Married women 15–49	Southern Africa
Benin	2018	11,168	5,338	3,607	2,535	1,072	DHS	Married women 15–49	West Africa
Burkina Faso	2010	13,563	5,520	3,323	2,414	909	DHS	Married women 15–49	West Africa
Burundi	2017	9,782	5,703	2,905	1,575	1,330	DHS	Married women 15–49	East Africa
Cameroon	2018	9,792	4,152	2,252	1,469	793	DHS	Married women 15–49	West Africa
Central African Republic	1995	4,083	1,380	780	559	216	DHS	Married women 15–49	Central Africa
Chad	2015	13,263	3,793	3,037	2,533	504	DHS	Married women 15–49	Central Africa
Comoros	2012	3,261	1,686	1,053	776	280	DHS	Married women 15–49	East Africa
Congo	2012	6,289	3,968	1,157	924	226	DHS	Married women 15–49	Central Africa
Congo Democratic Republic	2014	12,096	5,818	3,351	2,504	835	DHS	Married women 15–49	Central Africa
Cote d'Ivoire	2012	6,309	2,858	1,710	1,237	473	DHS	Married women 15–49	West Africa
Eswatini	2007	2,062	1,555	509	138	373	DHS	Married women 15–49	Southern Africa
Ethiopia	2016	10,223	5,950	2,280	1,329	951	DHS	Married women 15–49	East Africa
Gabon	2012	4,475	2,578	1,186	837	345	DHS	Married women 15–49	Central Africa
Gambia	2020	7,525	3,243	1,821	1,400	421	DHS	Married women 15–49	West Africa
Ghana	2014	5,321	3,022	1,591	926	665	DHS	Married women 15–49	West Africa
Guinea	2018	7,727	2,542	1,708	1,190	510	DHS	Married women 15–49	West Africa
Kenya	2014	18,549	14,004	3,246	1,707	1,540	DHS	Married women 15–49	East Africa
Lesotho	2014	3,612	2,839	665	307	358	DHS	Married women 15–49	Southern Africa
Liberia	2020	5,386	3,145	1,799	1,104	695	DHS	Married women 15–49	West Africa
Madagascar	2009	12,039	7,091	2,287	1,228	1,059	DHS	Married women 15–49	Southern Africa
Malawi	2016	16,130	12,565	3,016	1,742	1,274	DHS	Married women 15–49	Southern Africa
Mali	2018	8,567	3,521	2,048	1,439	608	DHS	Married women 15–49	West Africa
Mozambique	2011	9,332	4,685	1,530	1,530	625	DHS	Married women 15–49	Southern Africa
Namibia	2013	3,121	2,297	546	284	262	DHS	Married women 15–49	Southern Africa
Niger	2012	9,881	2,954	1,581	1,314	267	DHS	Married women 15–49	West Africa
Nigeria	2018	29,090	10,327	5,498	3,520	1,978	DHS	Married women 15–49	West Africa
Rwanda	2015	6,982	5,041	1,320	747	580	DHS	Married women 15–49	East Africa
Sao Tome and Principe	2009	1,718	1,306	646	309	337	DHS	Married women 15–49	Central Africa
Senegal	2019	10,895	4,195	2,364	1,765	588	DHS	Married women 15–49	West Africa
Sierra Leone	2019	9,714	4,478	2,409	1,690	719	DHS	Married women 15–49	West Africa
South Africa	2016	3,050	2,120	454	186	268	DHS	Married women 15–49	Southern Africa
Tanzania	2016	8,210	4,967	1,814	1,273	542	DHS	Married women 15–49	East Africa
Togo	2014	6,281	3,360	2,110	1,369	741	DHS	Married women 15–49	West Africa
Uganda	2016	11,223	7,553	3,187	2,054	1,134	DHS	Married women 15–49	East Africa
Zambia	2018	7,648	5,300	1,507	925	581	DHS	Married women 15–49	Southern Africa
Zimbabwe	2015	6,151	4,749	640	369	271	DHS	Married women 15–49	Southern Africa

### Data analysis

This study was based on secondary data analysis. Meta-analyses were performed with MetaXL application (Version 5.3, EpiGear International Pty Ltd, QLD, Australia). MetaXL tool is a user-friendly Microsoft Excel application used to perform meta-analysis. Meta-analysis is a term that refers to a set of statistical approaches for examining the impacts of a relationship between an independent and a dependent variable. Meta-analysis allows researchers to assess a wide range of effect sizes and heterogeneity across many studies. A forest plot and supporting statistics, like as confidence and prediction intervals, are used to display the basic outcomes of meta-analyses. Additional analyses performed by this statistical application tool include sub-group analysis and publication bias [24, 27, 28]. Meta-analyses are useful because they improve the generalizability of individual study results by providing a more exact estimate of the effect size [27]. Meta-analyses can improve statistical power and produce research results that can support generation of information to inform evidence-based decisions for policy and programme improvement [24].

In this study, the overall prevalence and its associated 95% confidence intervals (CI) for pooled and unmet needs, unmet needs due to limiting and spacing were calculated. Country‐specific estimates were also calculated and pooled using the Inverse Heterogeneity model (IVhet) meta‐analysis to yield 37 country estimates of FP indicators [27]. The inverse variance heterogeneity (IVhet) model is meta-analyse technique which is used to assess heterogeneity trends across studies. To account for study heterogeneity, the IVhet model generates a pooled estimate with a substantially broader confidence interval. The IVhet model maintains a correct coverage probability at a lower detected variance [28]. Sub-regional (West Africa, Central, East Africa or Southern Africa) and time stratified in 5 years periods (periods during which DHS data is collected) prevalence was also pooled.

Forest plots were used to display the estimated prevalence in individual countries and the pooled prevalence in each sub-region and its associated 95% confidence intervals (CI). Heterogeneity was quantitatively evaluated with the I^2^ statistic while publication bias was assessed using the Luis Furuya–Kanamori (LFK) index of the Doi plot [27]. Publication bias was examined to assess the extent to which the studies accurately measured the effect sizes of family planning indicators. A symmetrical Doi plot indicates that there is no reason to infer publishing bias, whereas an asymmetrical one does [24].

## Results

In total, 37 DHS datasets were eligible for final inclusion in the study, and these were drawn from four sub-regions of SSA (Fig. 1). The countries included 6 (16.2%) (Central African Republic, Chad, Congo, Congo Democratic Republic, Gabon and Sao Tome and Principe) from Central Africa, 7 (18.9%) (Burundi, Ethiopia, Kenya, Rwanda, Tanzania, Uganda and Comoros) from East Africa, 10 (27.0%) (Angola, Eswatini, Lesotho, Madagascar, Malawi, Mozambique, Namibia, South Africa, Zambia and Zimbabwe) from Southern Africa and 14 (37.8%) (Benin, Burkina Faso, Cameroon, Cote d'Ivoire, Gambia, Ghana, Guinea, Liberia, Mali, Niger, Nigeria, Senegal, Sierra Leone, Togo) from West Africa. Furthermore, 13.5% (*n* = 5) had their latest DHS conducted between 1995/6–2010 cycle while 40.5% (*n* = 15) and 45.9% (*n* = 17) had their latest DHS conducted between 2011–2015 and 2016–2020 cycles, respectively.

### Prevalence of unmet needs

**Fig. 2:**
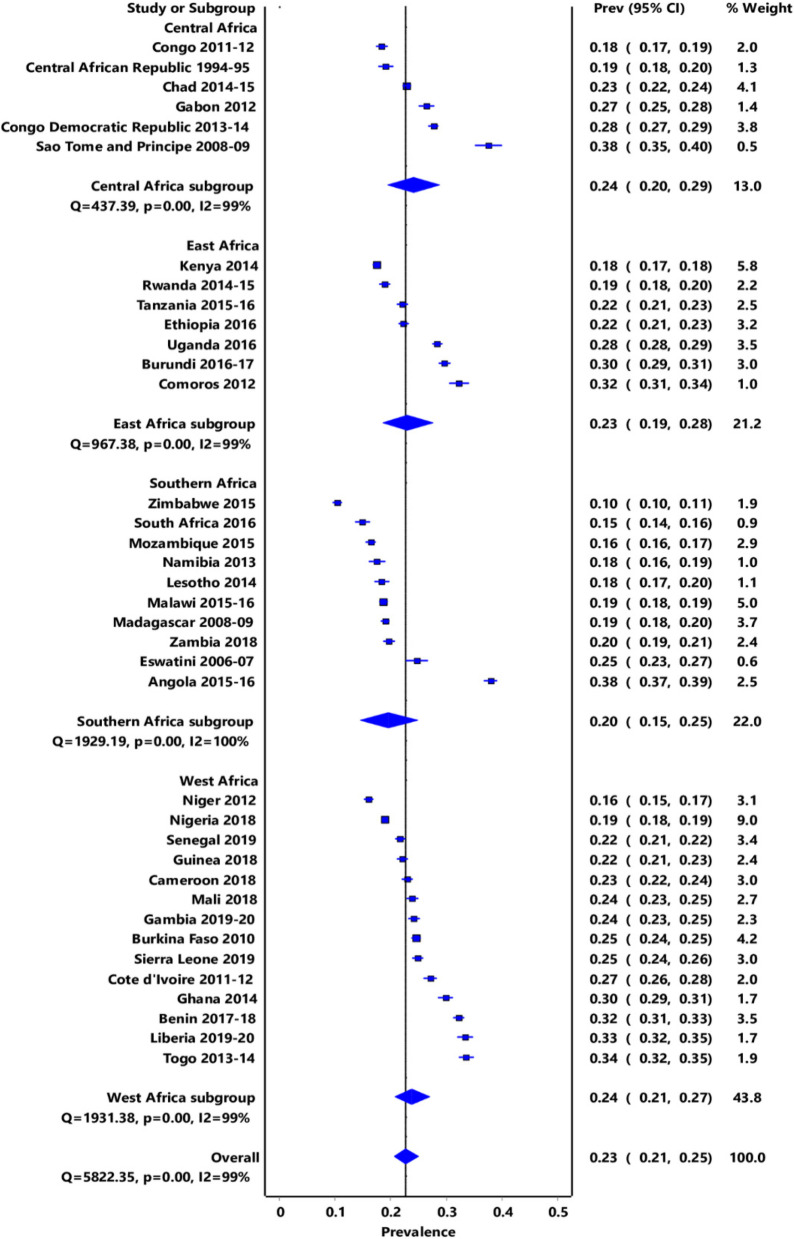
Country and regional prevalence of overall unmet needs for the 37 countries in SSA

The overall pooled prevalence of unmet needs for family planning for all 37 countries in the four sub-regions of SSA was 22.9% (95% CI: 20.9–25.0). The prevalence varied across countries from 10% (95% CI: 10–11%) in Zimbabwe to 38% (95% CI: 35–40) and 38 (95% CI: 37–39) (I^2^ = 99.8% and *p*-value < 0.0001) in Sao Tome and Principe and Angola, respectively. Twenty-four countries had the prevalence of unmet needs for family planning approximately 20% and above. Of these, 4 countries (Congo Democratic Republic, Gabon and Sao Tome and Principe) were from Central Africa, 5 countries (Burundi, Ethiopia, Tanzania, Uganda, and Comoros) were from East Africa, 2 countries (Eswatini and Zambia) were from Southern Africa and 12 countries (Benin, Burkina Faso, Cameroon, Cote d'Ivoire, Gambia, Ghana, Guinea, Liberia, Mali, Senegal, Sierra Leone, Togo) were from West Africa (Fig. 2). The total population of women who are married or in union aged 15 to 49 years from the 37 countries included in this analysis was 322,475. The country and regional prevalence of unmet needs for the 37 countries is illustrated in Fig. 2.

### Prevalence of limiting and spacing

The prevalence of unmet needs for family planning for limiting was 8% (95% CI: 7–9). The highest prevalence of unmet needs for limiting among the four sub-regions and within regional countries was observed in East Africa (Prev: 9%; 95% CI: 8–11) with Burundi having the highest prevalence (Prev: 14%; 95% CI: 13–14) while Kenya had the least (Prev: 8%; 95% CI: 8–9). Regionally, the lowest prevalence of unmet needs due to limiting was observed in Central Africa (Prev: 6%; 95% CI: 3–9) (Table 2). Overall, Niger (Prev: 3%; 95% CI: 2–3) had the lowest prevalence of unmet needs due to limiting while Sao Tome and Principe (Prev: 20%; 95% CI: 18–22) had the highest. The study further observed that the prevalence of unmet needs for family planning for spacing was 15% (95% CI: 14–17). The lowest prevalence of unmet needs for spacing among the regions was observed in Southern Africa (Prev: 12%; 95% CI: 8–16) while the highest was observed in Central Africa (Prev: 18; 95% CI: 16–21) (Table 2). Overall, Zimbabwe (Prev: 6% 95%; 5–7) had the lowest prevalence of unmet needs for spacing while Benin (Prev: 23%; 95% CI: 22–23) had the highest. A high level of heterogeneity (I^2^ = 99%) was observed. Furthermore, there was no publication bias and the Doi plot shows an LFK index of (0.79) which indicates no asymmetry. This means that there were significant differences in the prevalence of unmet needs across countries and sub-regions in SSA. (Supplementary file 3).

**Table 2:** Prevalence of unmet need for spacing and limiting among married women by sub-region

Variables/Sub-region	Sample size	Prevalence (95% CI)	I^2^	*P*-value
**Unmet needs for spacing**				
Central Africa	41,924	6 (3, 9)	99	0.00
East Africa	68,230	9 (8, 11)	98	0.00
Southern Africa	77,250	8 (7, 10)	98	0.00
West Africa	115,729	7 (6, 9)	99	0.00
**Unmet needs for limiting**				
Central Africa	41,924	18 (16, 21)	97	0.00
East Africa	68,230	14 (10, 17)	99	0.00
Southern Africa	77,250	12 (8, 16)	100	0.00
West Africa	115,729	17 (15, 19)	98	0.00

### Prevalence of unmet needs over time

**Fig. 3:**
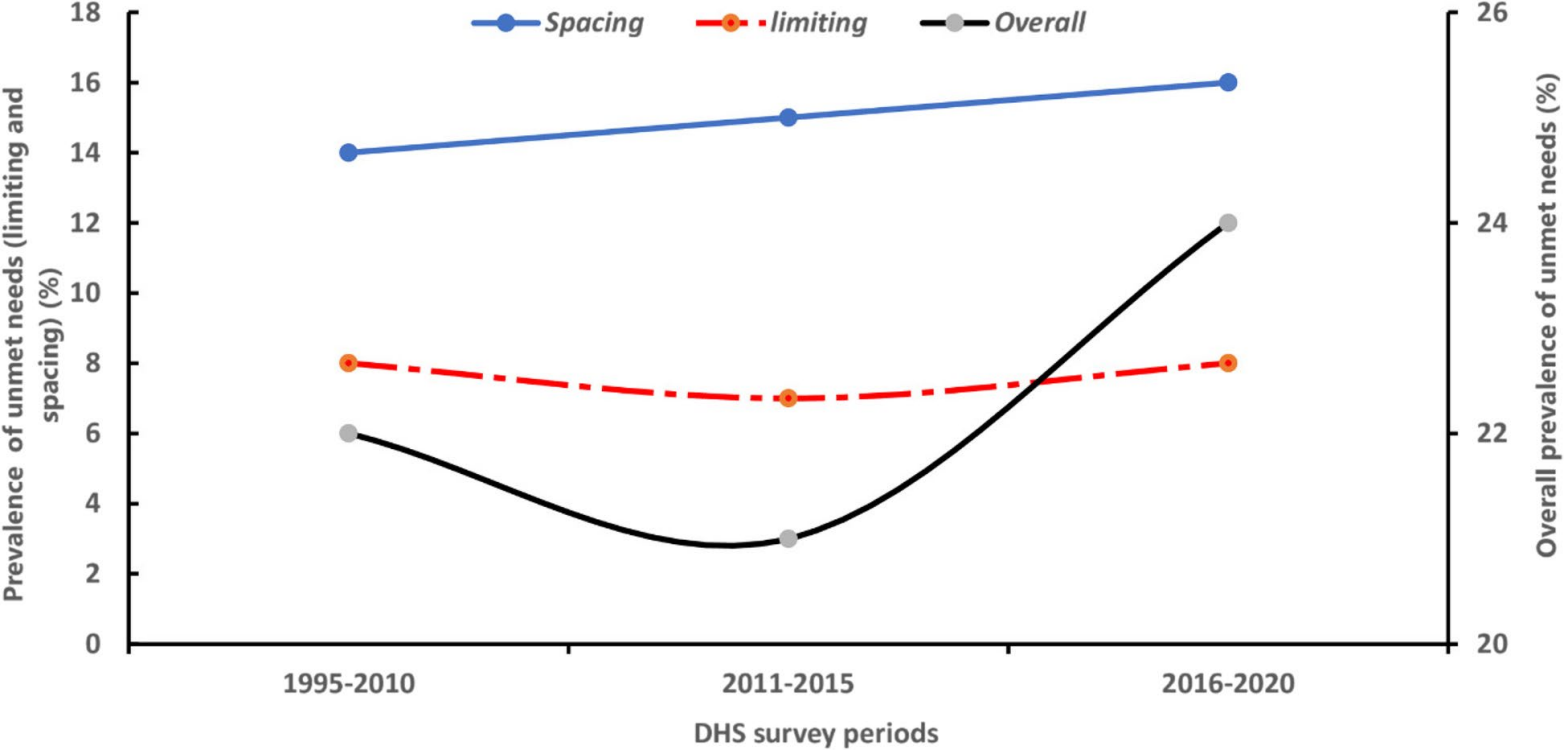
Country and regional prevalence of unmet need for family planning in sub-Saharan Africa by DHS period

The overall prevalence of unmet needs for family planning showed a decrease between the 1995/10 and 2011/15 waves. However, this decrease was not sustained as the prevalence increased from 21% (95% CI: 21–25) observed between 2011–2015 to 24% (95% CI: 21–27) in the 2016–2020 cycles. This trend was similar to the trend in the unmet needs due to limiting. A decrease in prevalence from 8% (95%: 4–13) to 7% (95% CI: 7–8) in 1995/10 and 2011/15 waves while in 2016–2020, there was an increase of 1% to that observed in 201/15 cycles. On the other hand, the prevalence of unmet needs for family planning due to spacing showed a 1% increase across the three periods. Between the 1995/10 and 2011/15 waves, the prevalence increased from 14% (95% CI: 9–19) to 15% (95% CI: 12–18) and later to 15% (95% CI: 13–18) in 2016–2020 cycles (Fig. 3).

## Discussion

The prevalence of unmet need for family planning is an important metric for designing family planning programs and has implications on maternal and child health. Previous studies have heightened progress in the implementation of family planning at a regional scale [29] however, there are risks of underestimation of the overall prevalence of unmet need for family planning due to masking of sub-regional differences. The current study set out to estimate the prevalence of unmet needs for family planning in women of reproductive age who are married or in a union in the four sub-regions of SSA. Our findings have shown variations in both unmet needs for family planning across the four regions of SSA. This can be attributed to differences in socio-cultural beliefs and practices, traditional and religious beliefs that prevent acceptance of family planning [30, 31].

Our results also suggest that ending unmet needs for FP by 2030 especially in SSA as set by the United Nations Population Fund (UNFPA) [32] may be far off unless deliberate policy re-alignment efforts are done. There is a need to improve access and quality of family planning services, enhance monitoring and follow-up of the progress on FP programmes and integration of FP in maternal health programmes. The regional variations in unmet needs for FP observed in our study may also be attributed to regional specific factors relating to social, cultural beliefs and religious beliefs [33]. For instance, in some religious circles, it is considered immoral and sinful to use family planning [30], thus influencing contraceptive decisions.. The observed low prevalence of unmet needs in Southern Africa may be owing to effective design and successful implementation of SRH policies and programmes which have seen increased demand for family planning services in the region [34].

Despite several efforts and initiatives that governmental and non-governmental organizations have implemented in most countries in SSA, our study shows that levels of unmet needs for FP increased between the 2011–2015 to 2016–2020 DHS cycles. This could be attributed to a lack of family planning information among women of reproductive age in Sub-Saharan Africa, which has been necessitated by persistently low educational levels [35, 36]. Our study has compared levels of unmet needs for FP across different DHS cycles as a way of determining the progress that had been made by various strategies implemented to address the problem. The increase in the prevalence of unmet needs during the 2016–2020 cycle suggests the need for increased programme monitoring and tracking of success or achievements on an annual basis. An earlier study by Cleland et al. [37] concluded that countries in sub-Saharan Africa had the greatest need to address unmet needs due to high demand for family planning among women of reproductive age. Our results further suggest the need to enhanced efforts by building on past and present successful interventions to reduce the unmet needs for FP to achieve the SDG targets 3.7.1 and goal 5, to reduce maternal and child morbidity and mortality [4, 38–40]. Reducing the gap in unmet needs goes beyond improving maternal and child health to improving the household and community levels economic status. For instance, reducing unmet needs leads to spaced children, increased investment in maternal and child education, acquisition of life skills which may be essential in income generation [26]. There is a need therefore to emphasize the need to reduce unmet need for FP through enhanced health education and improving access and choices of FP methods.

To help reduce the unmet need for FP in SSA, various initiatives such as the FP2020 by the Bill and Melinda Gates Foundation, the UK Department for International Development, the United States Agency for International Development (USAID), and the United Nations Population Fund [10, 11] have been implemented. These programmes have been supplemented by efforts from UNFPA and UNICEF to support national health systems through a steady, reliable supply of quality contraceptives [22]. The differences in the country-level prevalence of unmet needs for FP as observed in our study suggest the need for governments to ensure a continuous supply of contraceptives and increasing accessibility to sexual and reproductive health services especially among the hard-to-reach populations and adolescents. According to Montoya et al. [41], increasing access to contraceptives reduces maternal mortality as it decreases the risks of unsafe abortions and unintended pregnancies which account for the majority of maternal deaths especially in countries with high unmet needs for FP [42].

The main strength of the study is that it included at least one available latest DHS dataset from all countries with DHS in sub-Saharan Africa. While some countries had not conducted DHS for some time, their country estimates were essential in understanding the trends that have been achieved in reducing the prevalence of unmet needs for FP. However, some of the limitations stemming from the study are that not all countries in SSA had accessible DHS data. However, estimation of the prevalence based on the sub-regions offers a basis for approximation of unmet needs in SSA. Notwithstanding the limitations, our results have underlined huge variation in both country level and sub-regional level prevalence of unmet needs thus generating needs to learn from within the sub-regions and among countries such as Ethiopia, Kenya, Rwanda, and Zimbabwe that have run successful family planning programs [43].

## Conclusion

Our study highlights the need to improve the availability and accessibility of contraceptives to increase demand and reduce the unmet need. There is need to re-evaluate current FP strategies in the SSA and design new strategies to resolve the problem. Building on past successful programmes, the new strategies should ensure a continuous supply of contraceptives to reduce unmet needs for family planning and associated maternal and child health outcomes in sub-Saharan Africa. There is need for further research to understand best practices regarding SRH programming from better performing countries. The documented practices can be adapted by countries with high unmet needs to improve their situation.

## Supplementary Information

**Additional file 1: Supplementary file 1. **Forest plot of regional prevalence of unmet needs due to limiting for the 37 countries in SSA. **Supplementary file 2. **Forest plot of regional prevalence of unmet needs due to spacing for the 37 countries in SSA. **Supplementary file 3. **Doi plots assessing publication bias (a) Overall unmet needs (b) unmet needsdue to limiting (c) unmet needs due to spacing.

## Data Availability

Data used in this study is readily available at DHS website (www.dhsprogram.com). Other materials such as Do-files can be shared upon request to the corresponding author.

## Abbreviations

CI: Confidence Interval
DHS: Demographic and Health Survey
EA: Enumeration Area
FP: Family Planning
SDG: Sustainable Development Goal
SRH: Sexual Reproductive Health
SSA: Sub-Saharan Africa
UN: United Nations
UNFPA: United Nations Population Fund
UNICEF: United Nations Children Fund
USAID: United States Aid for International Development
WHO: World Health Organisation
