# Supplementary material for: Unmet need for family planning among married women in sub-Saharan Africa: a meta-analysis of DHS data (1995 – 2020)
Source: Contracept Reprod Med. 2023 Jan 11;8:3. doi: 10.1186/s40834-022-00198-5 (PMC9832678; doi:10.1186/s40834-022-00198-5)
Supplement: Supplementary file 1 — Additional file 1: Supplementary file 1. Forest plot of regional prevalence of unmet needs due to limiting for the 37 countries in SSA. Supplementary file 2. Forest plot of regional prevalence of unmet needs due to spacing for the 37 countries in SSA. Supplementary file 3. Doi plots assessing publication bias (a) Overall unmet needs (b) unmet needsdue to limiting (c) unmet needs due to spacing. [file 40834_2022_198_MOESM1_ESM.docx]

**Supplementary files**

Supplementary file 1: Forest plot of regional prevalence of unmet needs due to limiting for the 37 countries in SSA


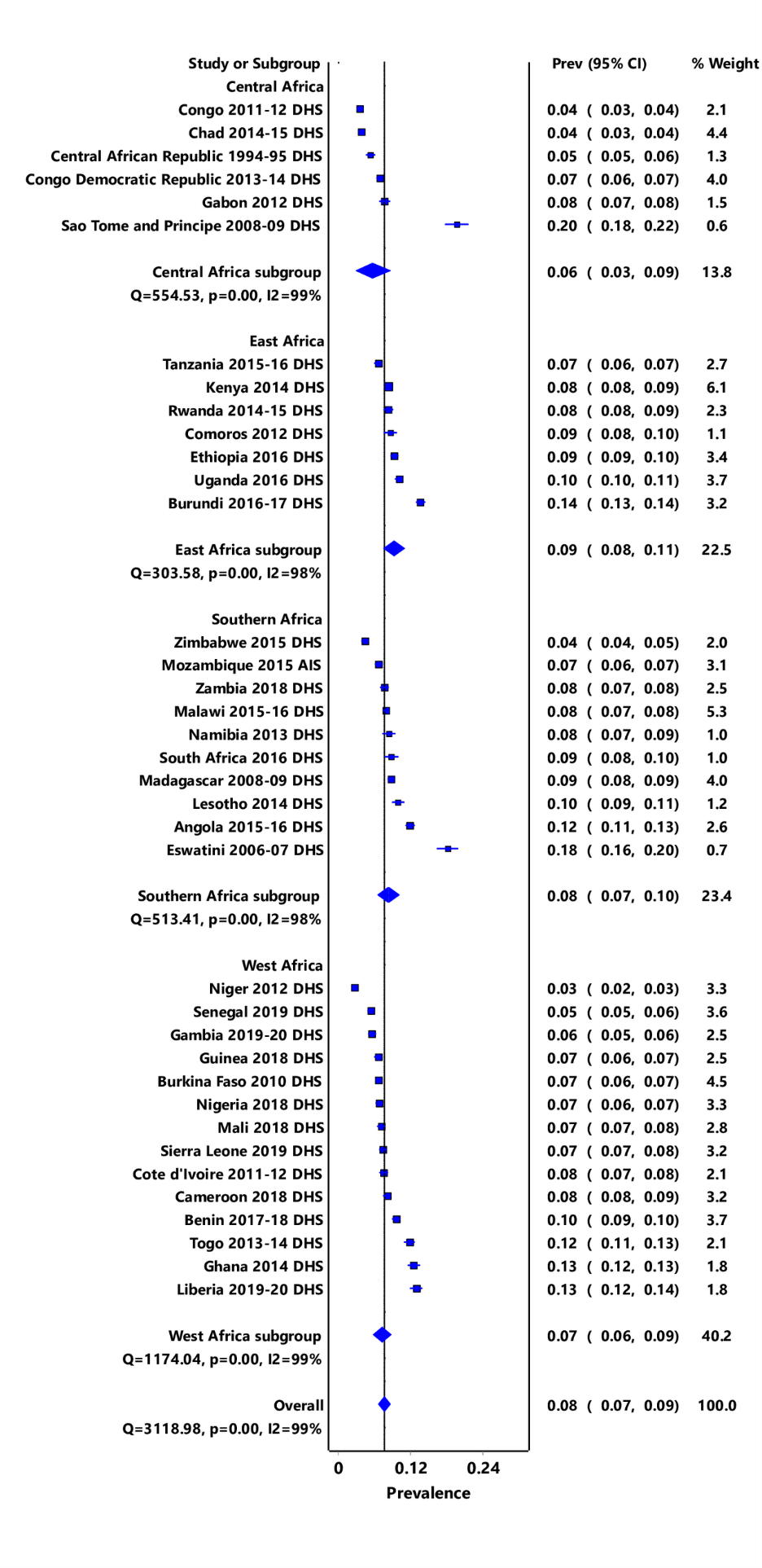


Supplementary file 2: Forest plot of regional prevalence of unmet needs due to spacing for the 37 countries in SSA


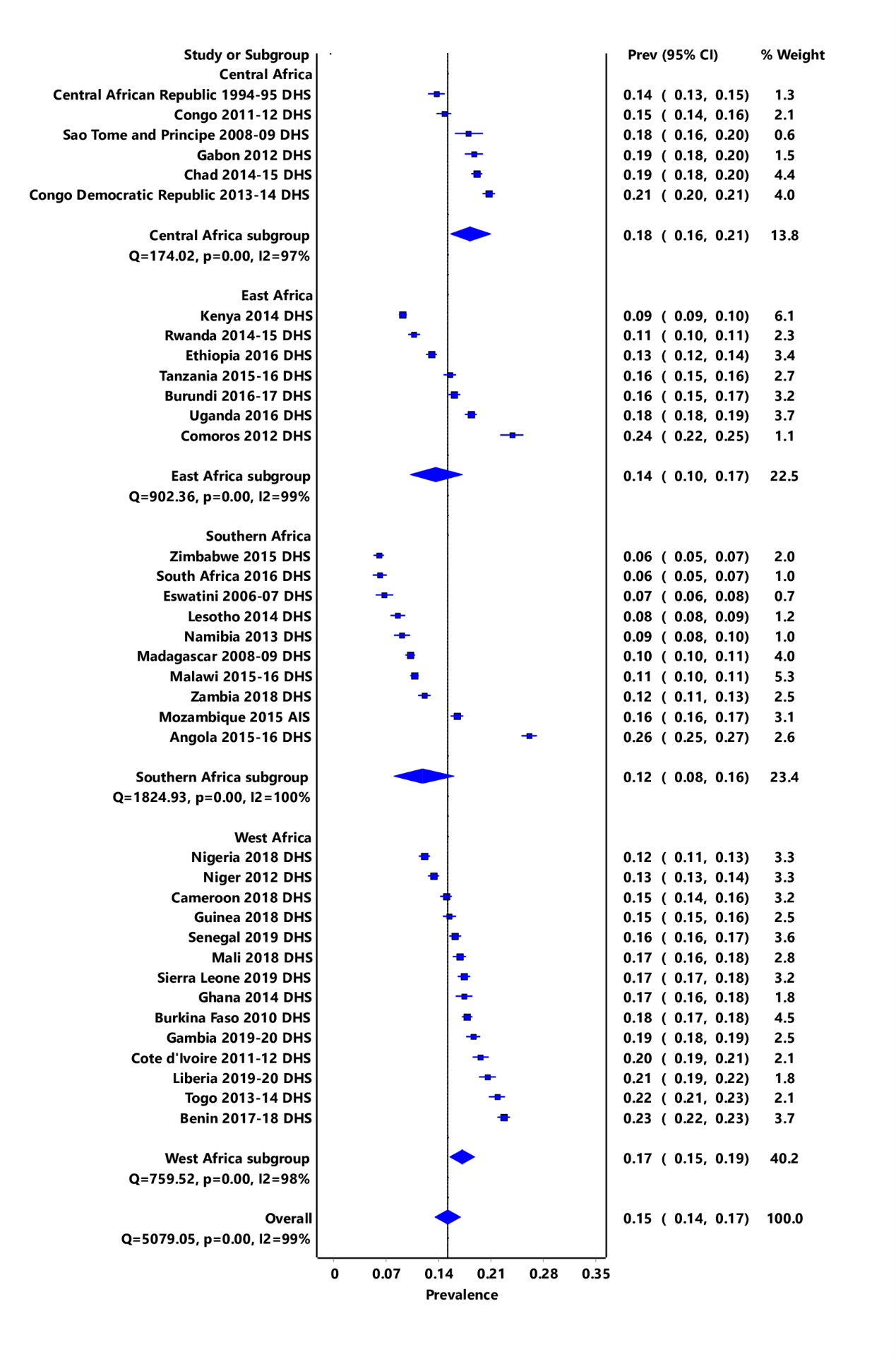


Supplementary file 3: Doi plots assessing publication bias (a) Overall unmet needs (b) unmet needs due to limiting (c) unmet needs due to spacing.
